# Decreased Naïve T-cell Production Leading to Cytokine Storm as Cause of Increased COVID-19 Severity with Comorbidities

**DOI:** 10.14336/AD.2020.0619

**Published:** 2020-07-23

**Authors:** Michael D Schwartz, Stephen G Emerson, Jennifer Punt, Willow D Goff

**Affiliations:** ^1^Morsani College of Medicine, University of South Florida, Tampa, Florida, USA; ^2^Immunology and Medicine, Columbia University, New York, NY 10027, USA; ^3^Immunology, Pathobiology, University of Pennsylvania School of Veterinary Medicine, PA 19104, USA; ^4^Colgate University, Hamilton, NY 13346, USA

**Keywords:** COVID-19, SARS-CoV-2, naïve T cells, type 2 diabetes, cytokine storm

## Abstract

Aging, type 2 diabetes, and male gender are major risk factors leading to increased COVID-19 morbidity and mortality. Thymic production and the export of naïve T cells decrease with aging through the effects of androgens in males and in type 2 diabetes. Furthermore, with aging, recovery of naïve T-cell populations after bone marrow transplantation is delayed and associated with an increased risk of chronic graft vs. host disease. Severe COVID-19 and SARS infections are notable for severe T-cell depletion. In COVID-19, there is unique suppression of interferon signaling by infected respiratory tract cells with intact cytokine signaling. A decreased naïve T-cell response likely contributes to an excessive inflammatory response and increases the odds of a cytokine storm. Treatments that improve naïve T-cell production may prove to be vital COVID-19 therapies, especially for these high-risk groups.

We are interested in the recent article by Fulzele et al. They wrote about the role of the profile and abundance of host cellular micro RNA in the immune response to SARS-CoV-2 [1]. We are intrigued by the proposition that diminished micro RNA response with aging might underlie increased severity of COVID-19. Severe SARS-CoV-2 and SARS infections are notable for severe T-cell depletion [2]. A decreased naïve T-cell response that allows more SARS-CoV-2 viral infection and replication likely contributes to subsequent uncontrolled cytokine production and clinical illness severity.

Multiple components of host immunologic response to pathogens are diminished with aging [3]. The thymus undergoes age-associated involution, with studies showing thymic size decreasing from birth at a rate of approximately 3% per year until middle age, and at a rate of 1% per year thereafter. Although the thymus continues to generate functional T cells, there is an age-related decline in naïve thymic T cell export [4]. We propose that decreased naïve T-cell production is a key component of COVID-19 severity with aging.

The signal joint/beta arrangements (sj/β) ratio is a signature of the recent thymic emigrant component of peripheral T-cell pool. The sj/β ratio persists at approximately 100:1 in young people. During senescence, the sj frequencies decline. But until age 70, DβJβ species remain unchanged. The resulting decrease in the sj/β ratio, which falls below a value of 1:10, indicates dramatically lower thymic production of naïve T cells.

T cells from older people display a particular propensity for oligoclonal expansion, which skews the T-cell repertoire toward antigen specificities previously encountered. Consequently, an expansion of terminally differentiated memory T cells upholds the cellularity of the peripheral T-cell pool. As a result of this compensatory mechanism and the progressive decline in thymic function, the composition of the peripheral T-cell pool is progressively altered, diminishing the ability to respond to a novel viral infectious challenge.

The COVID-19 mortality rate increases substantially in the eighth decade of life. Consistent with this hypothesis, there is further impairment of T-cell neogenesis after age 70 when DβJβ-rearrangement also decreases [5].

Studies of the reconstituted immune system after allogenic bone marrow transplant further highlight the decline of naïve T-cell production with aging and its direct relation to excessive inflammation. Naïve T cells are generated from hematopoietic progenitors through a thymus-dependent regenerative mechanism. Recent thymic emigrants expand in the periphery in response to homeostatic signals and after stimulation by their cognate antigen. Under physiologic conditions, thymic export assures a constant and lifelong supply as naïve T cells harbor a diverse T-cell receptor repertoire. Among the many studies linking transplant recipient age to increased graft vs. host immunopathology, one study in children and young adults showed that all recipients of an unrelated T-cell depleted marrow transplant (median, 12 years; range, 2-19 years) generated normal naïve CD4+ T-cell numbers within a year after transplantation. In contrast, the rebuilding of the T-cell compartment in adult recipients (median, 38 years; range, 20-59 years) was only completed after 2 to 3 years [6]. Furthermore, chronic graft vs. host disease, which is more common with older recipients, is associated with marked impairment in naïve T-cell production compared to patients who do not develop this complication [7].

In addition to greater mortality with aging, COVID-19 is also uniquely associated with a dramatic increase in morbidity and mortality in males and type 2 diabetics. Notably, studies have shown that naïve T-cell immune response deteriorates more rapidly in men than in women. Type 2 diabetes is also associated with decreased naïve T-cell production [8, 9]. We propose that decreased naïve T-cell function is a key component of COVID-19 severity in concert with these comorbidities.

Thymic involution is more rapid in males, likely due to an androgen effect. Pido-Lopez et al. quantified recent thymic emigrants (RTEs) in human blood through the measurement of sjTRECs. They found a decline in RTE numbers in the blood with increasing age and discerned that this decline is gender-linked and hastened in men. Peripheral blood from females contained significantly higher levels of sjTRECs per CD3+ T cell than blood from males (P = 0·002) without significant gender difference in the absolute number of CD3+ T cells in the populations analyzed (P > 0.10) [7, 8].

The medical profession and the general public widely accept that diabetes patients have an increased propensity for infections. Neutrophil chemotaxis and adherence to vascular endothelium, altered cytokine production, phagocytosis, intracellular bactericidal activity, opsonization, and cell-mediated immunity are all depressed in diabetics. Dworacki et al. profiled thymic output in type 2 diabetics by sjTREC analysis and evaluated naïve T -cell content in peripheral blood by assessing CD127 and/or CD132 antigen expression. They found that recent thymic emigrants and naïve CD127+ CD132+ cell populations were less numerous than in non-diabetic participants [9].

Optimal blood sugar control (mean hemoglobin A1c 7.3%) compared with sub-optimal control (mean hemoglobin A1c 8.1%) dramatically decreased COVID-19 in hospital mortality for patients with type 2 diabetes from 11% to 1.1% [10]. Since metformin treatment of type 2 diabetes—but not metformin combined with insulin treatment—increased recent thymic emigrants and naïve T-cell populations [9], perhaps that benefit is greater with metformin than other type 2 diabetes treatments? This area requires further study.

In COVID-19, suppression of initial interferon signaling but intact cytokine signaling by infected respiratory tract cells has been postulated to lead to a cytokine storm. This storm results in excessive neutrophilic and monocytic tissue destruction of lung tissue and damage to type 2 pneumocytes with resultant ARDS [11]. Consistent with this possibility, interferon treatment—which promotes apoptosis of T memory cells—specifically, nebulized interferon alpha2β alone, in addition to nebulized arbitol anti-viraI treatment, reduced the duration of detectable virus in the upper respiratory tract. In parallel, it reduced the duration of elevated blood levels of IL-6 and CRP [12]. Similarly, parental interferon combined with lopinavir, ritonavir, and ribavirin was shown to be superior to lopinavir and ritonavir with shortening of time to negative nasopharyngeal swab from 12 to 7 days [13]. The interferon-promoted apoptosis of preexisting memory T cells leads to rapid phagocytosis by CD8α+ DCs, and directly promotes the proliferation of antigen (Ag)-specific CD8 T cells at the beginning of the response. Furthermore, interferon indirectly enables latecomer Ag-specific T cells to become immediate effectors.

Recombinant interferon (IL)-7 is under investigation as an agent to increase thymic output of T cells. Simian IL-7 in aged rhesus monkeys resulted in increased numbers of naïve CD4 and CD8 cells. In a phase 1 trial, Perales et al. demonstrated that r-hil-7 (CYT107) promoted T cells in 12 patients after recovery from allogenic stem cell transplantation, including naïve T cells. IL-7 should be evaluated as an early treatment in COVID-19 illness [14].

An observational study in Italy suggested that men on androgen deprivation therapy for prostate cancer had a decreased risk of SARS-CoV-2 infection [15]. The authors postulated this was related to the effect of anti-androgens on ACE 2 receptors. However, castration in middle-age mature mice has resulted in rapid improvement of thymic function. This castration-induced rapid regeneration of the involuted thymus was associated with an increase in naïve CD4+CD8+ double positive thymocytes [16]. In human subjects, Goldberg et al. demonstrated that luteinizing hormone-releasing hormone (LHRH) enhances T-cell recovery after bone marrow transplantation, predominantly in the naïve T cell compartment [17]. Thus, the theory of anti-androgen benefit may be related to improved T-cell response instead of changes in ACE-2 receptor levels. Randomized studies of bicalutamide and degarelix in hospitalized patients with Covid-19 are enrolling as of publication.

In summary, we hypothesize that a decreased naïve T-cell response in the aging population, further diminished in men and type 2 diabetic patients, prevents successful inhibition of viral spread at an early stage of COVID-19 infection. This leads to an inappropriate inflammatory response that contributes to the morbidity and mortality observed in these groups. Treatments specific to these exacerbating conditions have been shown to improve T-cell response. The use of such agents may prove vital in preventing progression to a cytokine storm and ARDS.
